# Specific Physiological and Anatomical Traits Associated With Polyploidy and Better Detoxification Processes Contribute to Improved Huanglongbing Tolerance of the Persian Lime Compared With the Mexican Lime

**DOI:** 10.3389/fpls.2021.685679

**Published:** 2021-08-26

**Authors:** Gary Sivager, Leny Calvez, Saturnin Bruyere, Rosiane Boisne-Noc, Pierre Brat, Olivier Gros, Patrick Ollitrault, Raphaël Morillon

**Affiliations:** ^1^CIRAD, UMR AGAP Institut, Equipe SEAPAG, Petit-Bourg, Guadeloupe, French West Indies—UMR AGAP Institut, Univ. Montpellier, CIRAD, INRAE, Institut Agro, Montpellier, France; ^2^CIRAD UMR Qualisud Dpt PERSYST-Qualisud, Univ. Montpellier, Avignon Université, CIRAD, Institut Agro, IRD, Université de La Réunion, Montpellier, France; ^3^C3MAG, UFR des Sciences Exactes et Naturelles, Université des Antilles, Pointe-à-Pitre, Guadeloupe

**Keywords:** callose deposition, citrus, detoxification, huanglongbing, lime, triploid

## Abstract

Huanglongbing (HLB) is presently a major threat to the citrus industry. Because of this disease, millions of trees are currently dying worldwide. The putative causal agent is a motile bacteria belonging to *Candidatus* Liberibacter spp., which is transmitted by psyllids. The bacteria is responsible for the synthesis of callose at the phloem sieve plate, leading to the obstruction of the pores that provide connections between adjacent sieve elements, thus limiting the symplastic transport of the sugars and starches synthesized in leaves to the other plant organs. The Persian triploid lime (*Citrus latifolia*) is one of the most HLB-tolerant citrus varieties, but the determinants associated with the tolerance are still unknown. HLB-infected diploid Mexican lime (*Citrus aurantiifolia*) and Persian lime were investigated. The leaf petiole was analyzed using scanning electron microscopy (SEM) to observe callose deposition at the phloem sieve plate. Leaf starch contents and detoxification enzyme activities were investigated. In the field, Persian lime leaves present more limited symptoms due to HLB than the Mexican lime leaves do. Photosynthesis, stomatal conductance, and transpiration decreased compared with control plants, but values remained greater in the Persian than in the Mexican lime. Analysis of the petiole sieve plate in control petiole samples showed that pores were approximately 1.8-fold larger in the Persian than in the Mexican lime. SEM analyses of petiole samples of symptomatic leaves showed the important deposition of callose into pores of Mexican and Persian limes, whereas biochemical analyses revealed better detoxification in Persian limes than in Mexican limes. Moreover, SEM analyses of infected petiole samples of asymptomatic leaves showed much larger callose depositions into the Mexican lime pores than in the Persian lime pores, whereas biochemical traits revealed much better behavior in Persian limes than in Mexican limes. Our results reveal that polyploids present specific behaviors associated with important physiological and biochemical determinants that may explain the better tolerance of the Persian lime against HLB compared with the Mexican lime.

## Introduction

Huanglongbing (HLB) is one of the most devastating citrus diseases worldwide and is caused by a phloem-restricted gram-negative α-proteobacteria (*Candidatus* Liberibacter spp.; Bové, 2006). Three HLB-associated species were described in the taxonomy that were named according to their presumptive geographical origin: *Candidatus Liberibacter asiaticus* (Las) (Bové, 2006), *Candidatus Liberibacter africanus* (Jagoueix et al., 1994), and *Candidatus Liberibacter americanus* (Teixeira et al., 2005). In the Caribbean area, Las is the only strain that has been present since 2006 (Luis et al., 2009). The Asian citrus psyllid (*Diaphorina citri*), which efficiently spreads the disease, is the vector of Las. HLB affects tree development, fruit quality (Dala-Paula et al., 2019; Koh et al., 2020), and yields, which cause heavy economical loss (Tania Santivañez et al., 2013; Neupane and Moss, 2016) and can lead to tree death. A distinctive symptom of HLB is leaf yellowing with an asymmetrical pattern of blotchy yellowing or mottling of the leaf (Bové, 2006). Dieback of the canopy (Bové, 2006; Gottwald, 2010) follows the leaf symptoms. The disease causes relate to plant physiological perturbations that lead to increased callosis synthesis in phloem cells, which is responsible for plugging the sieve plate pores of the phloem cells (Achor et al., 2010; Koh et al., 2012; Albrigo and Stover, 2015), thus limiting symplastic transport between phloem cells. This callose deposition is often followed by phloem cell wall distortion, which may lead to sieve element collapse (Etxeberria and Narciso, 2015). Sugars and starch granules accumulate in the leaves (Schneider, 1968; Ed Etxeberria et al., 2009; Achor et al., 2010) because they can no longer be translocated to other organs. In the meantime, the starch and sugar contents in the symptom-presenting, HLB-infected leaves are much higher than in the controls (Achor et al., 2010; Fan et al., 2010). Such modifications of the leaf and root metabolisms (Bowman et al., 2016) have consequences, including deficiencies of microelements, such as zinc in the leaves and boron in the roots (Aubert, 2009). When infected, the tree is subjected to significant oxidative stress, leading to the induction of detoxification mechanisms that have crucial roles in disease adaptation (Martinelli et al., 2016).

It has long been considered that there is no genetic resistance to HLB in citrus. However, important variabilities of behavior under HLB constraints have been reported for the different species of the genus *Citrus* (Stover et al., 2014; Miles et al., 2017), which should be associated with better adaptation to the bacteria or differential attractiveness to the vector. For the related genera *Poncirus*, the lower susceptibility of trifoliate orange could be related to a lower leaf appetence of *D. citri*, which, in turn, would limit the possibilities of infection and development of Las (Westbrook et al., 2011; Richardson and Hall, 2013). More recently, the evaluation of HLB symptoms in germplasm collections under HLB constraints revealed complete resistance to HLB in related genera and particularly in Australian citrus species (Ramadugu et al., 2016; Alves et al., 2021). Several HLB-tolerant citrus hybrids and relatives were shown to synthetize antimicrobial peptides that can inhibit Las infections (Blaustein et al., 2018; Huang et al., 2021).

Within the genus *Citrus*, the triploid Persian lime (*Citrus* x *latifolia* Tan.) is one of the least susceptible to HLB (Gmitter et al., 2010; Evans et al., 2014). Recent work suggested that the tolerance of Persian lime is favored by a specific phloem regeneration mechanism that maintains the phloem sap flow (Deng et al., 2019). However, the phylogenetic origin and triploid level of this horticultural group should also be the major determinants of the better behavior under an HLB constraint. Persian lime results from the fusion between a diploid male gamete of *Citrus* x *aurantiifolia* (Christm.) Swing and a haploid female gamete of *Citrus* x *limon* L. (Burm; Ahmed et al., 2019, 2020). These two species are a result of interspecific hybridization between *Citrus medica* L. (citron) as the male parent and *C. x aurantium* L. and *Citrus medica* x *micrantha* Wester for *C. x lemon* and *C. x aurantiifolia*, respectively (Curk et al., 2016). Previous observations suggested that citron in the pedigree significantly correlated with HLB tolerance (Miles et al., 2017). As they both have a citron pedigree, the two varieties should have a greater tolerance to HLB. However, the Persian lime is the only genotype to present a greater tolerance to HLB compared to other cultivated genotypes (Evans et al., 2014).

Polyploidy is recognized as a major force driving plant evolution (Soltis and Soltis, 2009; Chen, 2010). Polyploidy is often associated with a wide range of morphological and physiological changes that may be advantageous under harsh environments (Brochmann et al., 2004). Indeed, several investigations have shown that polyploidization improves stress tolerance under adverse environmental conditions (for review, see Ruiz et al., 2020), which remains true in woody grafted plant species (Khalid et al., 2020; Ruiz et al., 2020). Most citrus are diploid, and several citrus species produce apomictic seeds favoring the natural occurrence of polyploidy among seedlings (Aleza et al., 2011). Interestingly, when subjected to varied stress, triploid (Lourkisti et al., 2020, 2021) and tetraploid citrus (Allario et al., 2013; Tan et al., 2015; De Souza et al., 2017; Oustric et al., 2017, 2019; Khalid et al., 2020) were more tolerant to oxidative stress because of more efficient oxidative systems. Recent observations suggest that some tetraploid rootstocks may limit the development of HLB (Grosser and Gmitter, 2011; Grosser et al., 2012). Reactive oxygen species (ROS) are free radicals and non-radical molecules, which are key components of the signaling pathway network and act as the main regulators of plant cell physiology and cellular responses to environmental factors (Bhattacharjee, 2012). Among them, hydrogen peroxide (H_2_O_2_) is an important, relatively stable, non-radical ROS. At low concentrations, H_2_O_2_ acts as a signal molecule involved in the regulation of specific biological and physiological processes (e.g., photosynthetic functions; cell cycle, growth, and development; and plant responses to biotic and abiotic stresses). Oxidative stress and eventual cell death in plants can be caused by excess H_2_O_2_ accumulation. As stress factors provoke an enhanced production of H_2_O_2_ in plants, severe damage can be induced at the cellular level. Plants are endowed with several H_2_O_2_-metabolizing enzymes and, primarily, with catalases (CAT) or ascorbate peroxidases (APX). CAT is a peroxisome-localized enzyme which do not require any reductant for catalyzing a dismutation reaction. APXs have a high affinity for H_2_O_2_ and utilizes ascorbate (AsA) as a specific electron donor (Sofo et al., 2015).

In the present work, we compared the behavior under the HLB constraint and the control condition of the Mexican diploid lime (*C. x. aurantiifolia*) and the Persian triploid lime, which are known for their contrasted tolerance to HLB, respectively. We performed investigations in asymptomatic (Las+AS) and symptomatic (Las+S) leaves of infected trees compared to the control to understand the disease development process better. Control (Las−) and asymptomatic samples were collected on trees grown in distinct insect-proof greenhouses. Infection was obtained after grafting-infected budwoods to determine the precise moment of the infection. Thus, it was possible to limit the impact of other environmental factors. Symptomatic samples were collected on trees naturally infected by psyllids in the field because, in the greenhouse, we rarely acquire symptomatic leaves. The physiological leaf behavior was evaluated by measuring leaf gas exchanges. In this article, we performed anatomical measurements using scanning electron microscopy (SEM) and fluorescent microscopy, which were associated with the investigation quantification of antioxidant molecules enzymes involved in detoxification processes. Taken together, our findings provide new knowledge on the determinant of the phenotypic differentiation between Mexican limes and Persian limes, which highlighted the importance of ploidy for HLB disease tolerance within the genus *Citrus*.

## Materials and Methods

### Plant Material and Growth Condition

The INRAE-CIRAD of San Giuliano in Corsica (France) provided diploid Citrumelo (SRA 928; *Citrus sinensis* [L.] Osbeck × *Poncirus trifoliata* [L.] Raf) seeds from the collection of the “CRB Citrus” biological resource center (Luro et al., 2017). Seedlings were planted in substrate and were kept in a greenhouse for 10 months. The ploidy status of 2× seedlings was checked and confirmed by flow cytometry (Partec I) according to Froelicher et al. (2007). Genetic conformity of the seedlings was confirmed by using SNPs markers developed by Bruyère et al. (2016).

Thirty genetically identical and uniform 2× seedlings were selected for further investigation. To compare the HLB tolerance of genotypes, controlling the development of the disease and transmitting the disease by grafting was necessary. Ten 10-month-old rootstock seedlings were grafted using budwoods collected near asymptomatic HLB leaves and control leaves of the 2× Mexican lime [*C. aurantifolia* (Christm. Swingle, SRA 140)] and the 3x Persian lime [*C. latifolia* (Yu. Tanaka) Tanaka, SRA 58]. The budwoods of asymptomatic HLB leaves were collected from a single tree initially infected in the greenhouse by using psyllids collected in the field. Budwoods of that tree tested molecularly positive for HLB. Inverted t-budding was performed 30 cm above the ground. Six control and infected trees of each genotype were grown in 40-L pots in close, but distinct, greenhouses for 18 months with day and night temperatures of 25–32°C and 22–28°C, respectively, and a relative humidity varying between 70 and 98%. These trees were used for the physiological, microscopic, and biochemical investigations.

Observations regarding the tree symptoms of HLB and a collection of symptomatic leaves (Las+S) were performed on the same 4-year-old rootstock and variety combinations planted in the field and naturally infected for 2 years.

### HLB Monitoring

DNA of *Candidatus Liberibacter asiaticus* was extracted from control leaves (Las−) and at different disease stages (Las+AS; Las+S) by using the Qiagen DNAeasy plant mini kit. The molecular detection of the bacteria was performed by using quantitative polymerase chain reaction (qPCR), according to Li et al. (2006), with the same primers and probes. Quantitative PCR were regularly performed (almost every 6 months) to characterize the infection status. After 18 months, all the trees presented a similar uniform size and were all positive for HLB.

### Leaf Physiological Parameters

The leaf thickness and area of each genotype were measured using a micrometer (IP65, Mitutoyo) and were scanned using Image J software.^1^ Leaf greenness was measured using a SPAD meter (Minolta, SPAD-502, Japan). Leaf stomatal conductance (*g*_*s*_), transpiration (*E*), and photosynthesis (*A*) were periodically measured with LCpro + Portable Photosynthesis Systems (ADC BioScientific Ltda., Hoddeston, United Kingdom). The intrinsic photosystem II quantum yield (QY), which is the equivalent to (F_*m*’_-F_0’_)/F_*m*’_, was checked using a leaf fluorometer (Fluorpen FP 100, Photos System Instrument, Czechia). Measurements were performed within light-acclimated leaves; thus, QY corresponds to the QY of antennas but not to the whole photosystem (Genty et al., 1989). The light pulse was set at 3,000 μmol photons m^–2^ s^–1^ (100%) to measure QY automatically according to the operation manual.

Each measurement (*g*_*s*_, *E*, *A, SPAD*, and QY) was taken between 9 and 11 am on 9–12 mature leaves selected at medium plant height. Measurements were then done using a photosynthetic photon flux density (PPFD) maintained at 1,400 μmol photons m^–2^ s^–1^.

Ten leaves of Mexican and Persian limes were used to measure their relative water contents (RWCs). Leaves were harvested in the morning at 9 am. The RWC (%) was determined as

$$\text{W}=100\times \left(\left[M_{\text{f}}-M_{\text{d}}\right]:\left[M_{\text{t}}-M_{\text{d}}\right]\right),$$

where *M*_f_ is the fresh mass; *M*_t_ is the turgid mass after leaf rehydrating; and *M*_d_ is the dry mass after drying the leaves in an oven. The leaf RWC takes into account the turgid mass of leaves. Thus, RWC corresponds to the proportion of the leaf water content related to the maximum water content contained in the leaf (Barrs and Weatherley, 1962).

Slides for the analyses of stomatal and epidermal cells and the number of stomata per unit of leaf surface area (stomatal density) were prepared by using the protocol developed by Morillon and Chrispeels (2001). Sixty stomata were measured from three different slide preparations that used three different mature leaves.

The starch content was estimated on leaves by using the method developed by Holm et al. (1986), with the absorbance measured at 510 nm.

### Petiole Preparation for SEM

Fresh cross sections of 2× (Mexican lime) and 3× (Persian lime) petioles were fixed overnight at 4°C in 2.5% glutaraldehyde in a phosphate saline buffer (pH 7.2). The sections were then dehydrated in a series of acetone solutions for increasing concentration until reaching 100% acetone. Samples were then dried to a critical point in CO_2_ and sputter-coated with gold before observation with a FEI Quanta 250 electron microscope at 20 kV. At least four independent preparations of three leaf petioles were used for each genotype for SEM analysis. These samples were also used for blue aniline staining preparations (see below). Some of the samples were also used for qPCR analysis (Table 1).

**TABLE 1:** Quantitative real-time PCR in the petioles of the control (Las−) as well as infected leaves that were asymptomatic (Las+AS) and symptomatic (Las+S) from Mexican and Persian limes, respectively.

	**Las**-		**Las+AS**		**Las+S**	
			
	**Mexican lime**	**Persian lime**	**Mexican lime**	**Persian lime**	**Mexican lime**	**Persian lime**
**Ct**	ND	ND	25.7 ± 0.4a	29.4 ± 0.4b	24.7 ± 0.2a	25.4 ± 0.7a

*Results are expressed as mean + SE (*n* = 3). For each given datum, different letters indicate a statistical difference between genotypes (one-way ANOVA followed by Tukey’s post hoc test, *P* ≤ 0.05).*

### Petiole Preparation for Blue Aniline Staining

Fresh cross sections of 2× and 3× petioles were fixed in formaldehyde overnight and then washed with a phosphate buffer (PBS, pH 7.2), followed by distilled water. The sections were dehydrated in a series of acetone solutions of increasing concentration and then incubated overnight in 100% acetone. The sections were analyzed after washing them quickly with phosphate buffer and staining for 10 min in an aqueous 0.1% blue aniline solution.

### Analysis of Stress Biomarkers and Detoxification System

All biochemical assays were performed by a Tecan Infinite 200 PRO machine and were done on control, asymptomatic, and symptomatic leaves. To evaluate the presence of stress biomarkers in both limes, the presence of Malondialdehyde (MDA) was determined according to the method described by Léchaudel et al. (2013). The first absorbance reading was performed at 532 nm and the second at 600 nm. The method used for H_2_O_2_ content estimation was adapted from Velikova et al. (2000), and the absorbance was read at 350 nm. Enzymes of the detoxification system were also dosed. The CAT assay was adapted from Aebi (1984), and the activity was estimated by Aebi (1984) by measuring the reaction rate at 240 nm for 20 min. An APX assay was performed according to Nakano and Asada (1981), with the measured activity at 290 nm. A total ascorbate (AsA) assay was performed according to the method by Kampfenkel et al. (1995). The absorbance was read at 525 nm.

### Fluorescence *in situ* Hybridization (FISH) of *Candidatus Liberibacter asiaticus* in the Petioles of Mexican and Persian Limes

For each sample, 330 g of petioles was ground in a 2-ml solution buffer (Na_2_HPO_4_/NaH_2_PO_4_; 0.1 M—pH 7.2). The ground samples were fixed in a final solution of 4% paraformaldehyde in the same buffer for 4 h at 4°C on silane-coated glasses placed in a wet chamber. Hybridization experiments were similar to those previously described by Brissac et al. (2011). Once the slides dried, the Cy3-marked target probe (Las406: 5′-CATTATCTTCTCCGGCG-3′) used in Hilf et al. (2013) was added for an overnight hybridization in a 20% formamide solution. Slides were counterstained by SYBR Green and mounted with Cytomation fluorescent mounting medium (Dakocytomation_@_, France) before visualization under an epifluorescence microscope (Eclipse 80i Nikon_@_, France).

### Statistical Analysis

For each given date of the experiment, data were subjected to variance analyses using a one-way ANOVA (SigmaPlot version 11, from Systat Software, Inc., San Jose, CA, United States).^2^ One-way ANOVA followed by Tukey’s *post hoc* test was used to assess significant differences. Statistical significance was set at *P* ≤ 0.05.

## Results

In the field, Persian lime trees show higher growth compared with Mexican lime trees: 4 years after planting and 2 years after natural HLB infection, tree height is approximately 30% higher. Leaf yellowing and leaf mottling are quite uniform on most Mexican lime leaves. In Persian lime trees, leaf symptoms (Las+S) with asymmetric yellow mottling can be observed on some twigs, but other twigs present leaves without any apparent symptom (Las+AS). The impact of HLB on leaf phenology is also different. The number of leaves is higher in the Mexican lime, but the leaf maintenance on the Persian lime twigs seems to be much longer. This result correlates with the presence of numerous dead Mexican lime leaves at the bottom of the trees.

**FIGURE 1:**
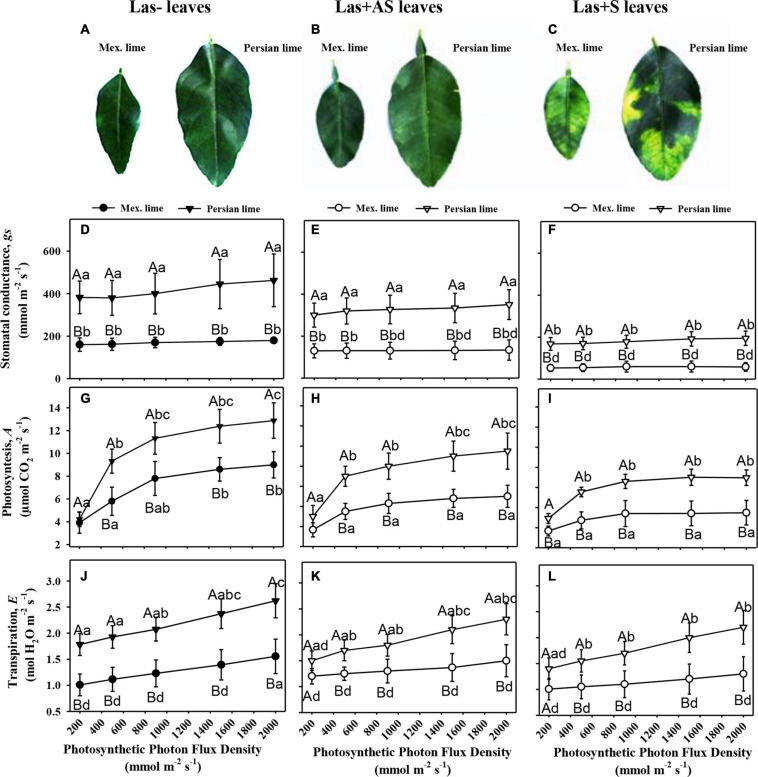
Physiological impact of HLB in infected leaves that were asymptomatic (Las+AS) and symptomatic (Las+S) compared with control (Las−) leaves from Mexican and Persian limes, respectively. Pictures of control **(A)**, Las+AS **(B)**, and Las+S **(C)** leaves of both cultivars. Stomatal conductance, gs **(D–F)**, photosynthesis, A **(G–I)**, and transpiration **(J–L)** measured in Las−, Las+AS, and Las+S leaves from Mexican and Persian limes, respectively, subjected to increased PPFD. The results are presented with mean values ± SE (*n* = 10). For each given datum, different letters indicate a statistical difference between genotypes (one-way ANOVA followed by Tukey’s *post hoc* test, *P* ≤ 0.05).

Control and infected trees grown in greenhouse conditions were tested by qPCR. As expected, controls were negative, and trees infected by HLB were positive (Table 1). Moreover, infected trees presented HLB symptoms. For Las+AS petiole leaves, the copy number of the bacterium quantified in Mexican lime trees was much higher than in Persian lime trees (Ct of 25.7 versus 29.4). In Las+S petiole leaves, the Ct values were similar for both genotypes (close to 25). These results are supported by fluorescence *in situ* hybridization (FISH) analysis using a probe targeted against Las on the same amount of grounded petiole material (Supplementary Figure 1). As expected, control samples did not present any fluorescence. However, the fluorescence in Mexican lime Las+AS was much higher than in Persian lime Las+AS (Supplementary Figures 1C,D). In Mexican and Persian lime Las+S petiole samples, the observed fluorescences were quite similar (Supplementary Figures 1E,F). Investigations were also performed at physiological, microscopic, and biochemical levels on the trees grown in greenhouse conditions. For control trees (Las−), the values of thickness, surface area, and RWC leaf values of Persian limes are significantly greater than those of the Mexican limes (Table 2). In addition, the stomatal size in the Mexican lime was smaller than in the Persian lime, which was the opposite of stomatal density, regardless of the plant’s sanitary status. The epidermal cell area followed the same trend as the stomata cell area. The leaf thickness values remained higher in Persian limes compared with Mexican limes. Finally, a decrease in SPAD, QY, and RWC values were observed in the Mexican Las + leaves compared with the Las− leaves. Gas exchange measurements were performed on leaves of Las− trees (Figure 1A), as well as on Las+AS (Figure 1B) and Las+S (Figure 1C) leaves of infected trees. For leaves of Las− trees, an increase in light intensity during measurements led to an increase in the measured values of the stomata conductance (gs; Figure 1D), of the photosynthesis (*A*; Figure 1G) and transpiration (*E;* Figure 1J); these parameters were always higher in Persian lime leaves compared with Mexican lime leaves. In Las+AS and Las+S leaves, the value increases observed in control (Figures 1G,J) due to the increasing light intensity are much more limited than in Las + AS and Las + S (Figures 1H,I,K,L). However, they remained consistently higher in Persian limes compared with Mexican limes (Figures 1F,I,L). Similar observations were also true for SPAD and QY (Table 2).

**TABLE 2:** Leaf physiological characterization of Mexican and Persian limes.

	Las-		Las+AS		Las+S	
			
	Mexican lime	Persian lime	Mexican lime	Persian lime	Mexican lime	Persian lime
**Thickness (mm)**	0.30 ± 0.06 a	0.41 ± 0.08 b	0.31 ± 0.07 a	0.45 ± 0.09 b	0.32 ± 0.05 a	0.54 ± 0.09 b
**Leaf area (mm^2^)**	1648 ± 340 a	3203 ± 695 b	1523 ± 280 a	3312 ± 825 b	1356 ± 312 a	3598 ± 1170 b
**SPAD (AU)**	57.2 ± 8.8 b	64.23 ± 8.61 b	55.2 ± 7.1 b	60.42 ± 8.6 b	41.5 ± 5.98 a	56.28 ± 11.94 ab
**QY (AU)**	0.61 ± 0.10 b	0.65 ± 0.07 b	0.62 ± 0.93 b	0.66 ± 0.12 b	0.36 ± 0.13 a	0.50 ± 0.13 ab
**RWC (%)**	80.4 ± 2.6 a	83.8 ± 0.7 b	79.2 ± 1.6 a	82.7 ± 2.6 b	76.2 ± 2.4 a	80.6 ± 3.2 a
**Stomata area (μm^2^)**	338 ± 7 a	360 ± 9 b	320 ± 12 a	370 ± 10 b	340 ± 13 a	366 ± 11 b
**Stomata density/mm^2^**	442 ± 7 b	381 ± 9 a	445 ± 6 b	390 ± 11 a	450 ± 10 b	401 ± 16 a
**Epidermal cell area (μm^2^)**	177 ± 5 b	210 ± 7 c	168 ± 4 b	221 ± 13 c	150 ± 8 a	230 ± 17 c

*Leaf thickness, leaf area, leaf greenness (SPAD unit), effective quantum yield of PSII (ΦPSII) within light-adapted leaves, relative water content (RWC), and leaf stomatal size and density, measured in the control (Las−) as well as infected leaves that were asymptomatic (Las+AS) and symptomatic (Las+S) from Mexican and Persian limes, respectively. Results are expressed as mean + SE (*n* = 6–100 measurements). ANOVA tests were performed to determine if HLB led to significant differences. For each given datum, different letters indicate a statistical difference between genotypes (one-way ANOVA followed by Tukey’s post hoc test, *P* ≤ 0.05).*

Measurements of starch content at the leaf level showed a higher amount in the Persian lime Las− leaves compared with the Mexican lime leaves (Table 3). In Las+AS leaves, a strong increase was found in the Mexican lime leaves compared with the control (about 10×), but it was only doubled in Persian lime leaves. For Las+S leaves, the starch content in Mexican limes and Persian limes are strong and not significantly different than for Las+AS Mexican limes (Table 3).

**TABLE 3:** Starch contents in the control (Las−) as well as infected leaves that were asymptomatic (Las+AS) and symptomatic (Las+S) from Mexican and Persian limes, respectively.

	Las-		Las+AS		Las+S	
			
	Mexican lime	Persian lime	Mexican lime	Persian lime	Mexican lime	Persian lime
**Starch (μg^–1^ DM)**	0.63 ± 0.01 a	1.30 ± 0.24 b	11.4 ± 0.03 d	2.13 ± 0.03 c	9.24 ± 0.10 d	10.85 ± 0.35 d

*Results are expressed as mean + SE (*n* = 3). ANOVA test was used to determine if HLB led to significant differences. For each given datum, different letters indicate a statistical difference between genotypes (one-way ANOVA followed by Tukey’s post hoc test, *P* ≤ 0.05).*

**FIGURE 2:**
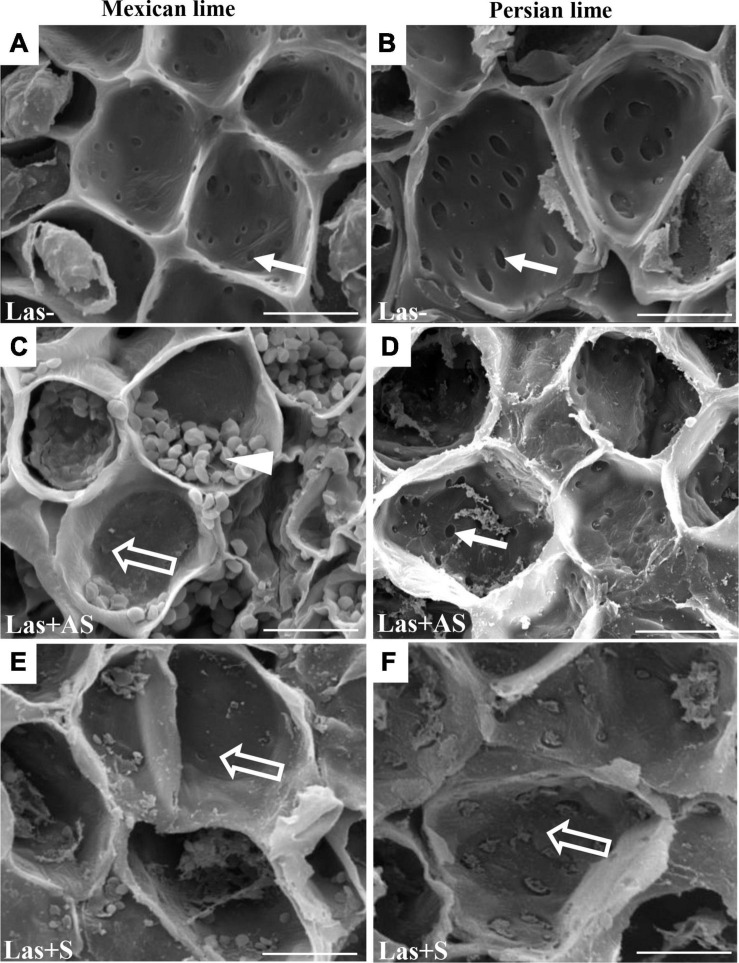
SEM of phloem of the control (Las−; **A,B**) as well as infected petioles from leaves that were asymptomatic (Las + AS; **C,D**) and symptomatic (Las + S; **E,F**) from Mexican and Persian limes, respectively. White arrows indicate pores not plugged by callose depositions, whereas opened arrows indicate pores with callose depositions. White triangle indicates the presence of starch grains. Bars = 30 μm.

**FIGURE 3:**
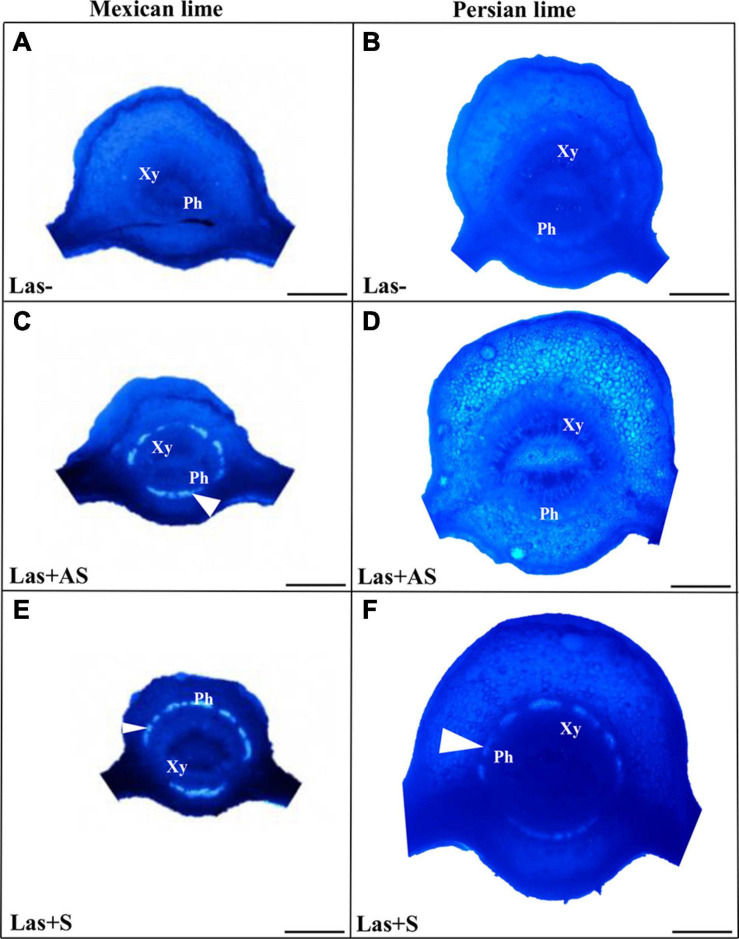
Aniline blue staining of the petioles of the control (Las−; **A,B**) as well as infected leaves that were asymptomatic (Las + AS; **C,D**) and symptomatic (Las + S; **E,F**) from Mexican and Persian limes, respectively. White triangles indicate the presence of callose deposition at the phloem location (Xy: xylem and Ph: Phloem). Bars = 0.5 mm.

To decipher the impact of the disease in the phloem, SEM analyses were performed in the leaf petioles of the different genotypes. The petiole diameters and areas were greater in Persian limes than in Mexican limes (Table 4). In Las+ samples, the cortex, the phloem and xylem areas, and the pith area were also greater in Persian lime compared with Mexican lime. For the Las− leaves, Mexican limes presented phloem cells and pores between cells that were smaller than in Persian limes (Table 4 and Figures 2A,B). For Las + leaf petiole samples, tissue and vessel collapses were observed. The pores in the petiole phloem cell walls of Las+AS Mexican lime leaves were no longer visible because they were clogged by callose deposits (Figure 2C). For Persian lime asymptomatic leaves, the pores were still not clogged, and no starch grain was present (Figure 2D). Interestingly, a large number of starch grains were visible in the petioles of Mexican lime Las+AS leaves (Figure 2C) but not in Persian lime leaves. For Mexican lime and Persian lime Las+S samples, pores were clearly plugged by callose deposits. Using the same type of samples, aniline blue staining was performed to quantify the callose deposits. No deposits were observed for the controls (Figures 3A,B). On the other hand, a white ring was clearly visible at the phloem location for Las+AS Mexican lime samples, which was not the case for the Persian lime samples (Figures 3C,D). For the Mexican lime and Persian lime Las+S samples, a callose ring was present in both genotypes, but the ring was more intense for Mexican limes (Figure 3).

**TABLE 4:** Anatomical characterization of leaf petioles of the control (Las−) as well as infected leaves that were asymptomatic (Las+AS) and symptomatic (Las+S) from Mexican and Persian limes, respectively.

	Las-		Las+AS		Las+S	
			
	Mexican lime	Persian lime	Mexican lime	Persian lime	Mexican lime	Persian lime
**Petiole diameter (mm)**	1.28 ± 0.10 a	2.56 ± 0.35 b	1.31 ± 0.12 a	2.98 ± 0.41 b	1.23 ± 0.20 a	2.78 ± 0.40 b
**Petiole area (μm^2^)**	1327714 ± 48027 a	4579717 ± 2107471 b	1000764 ± 76969 a	4799753 ± 1131552 b	1369740 ± 51821 a	5113210 ± 670576 b
**Cortex area (μm^2^)**	1292947 ± 83 a	1518156 ± 603498 ab	979552 ± 438032 a	2042778 ± 815231 b	954458 ± 428548 a	2036659 ± 911788 b
**Phloem area (μm^2^)**	223991 ± 33838 a	572203 ± 143981 b	178772 ± 57310 a	485768 ± 73518 b	177072 ± 25140 a	614605 ± 144724 b
**Phloem cells area (μm^2^)**	1341 ± 435 a	2485 ± 995 b	1046 ± 385 a	2374 ± 1281 b	1362 ± 629 ab	2845 ± 1225 b
**Pores of phloem cells area (μm^2^)**	14.4 ± 1.5 a	26.5 ± 9.0 b	–	11.2 ± 0.8 a	–	–
**Xylem area (μm^2^)**	251721 ± 27948 a	675409 ± 143965 b	192375 ± 56087 a	598872 ± 46435 b	199294 ± 36780 a	694855 ± 79640 b
**Pith area (μm^2^)**	93962 ± 33240 ab	116838 ± 17589 ab	50032 ± 3254 a	133796 ± 40519 b	58734 ± 200692 a	156156 ± 53880 b

*Results are expressed as the mean ± SE (*n* = 6–50). ANOVA tests were performed to determine if HLB led to significant differences. For each given datum, different letters indicate a statistical difference between genotypes (one-way ANOVA followed by Tukey’s post hoc test, *P* ≤ 0.05).*

Assays related to the quantification and the enzymatic activity of the main stress biomarkers and detoxification enzymes were performed by using leaf samples. A significant increase in MDA was observed in Las+AS Mexican lime leaves compared with the controls, but not in Persian lime leaves (Table 5). Increases in H_2_O_2_ and AsA contents were observed for Mexican lime and Persian lime Las+AS samples compared with controls. However, CAT activity increased in Persian lime Las+AS leaves, but it did not increase in Mexican lime leaves. Interestingly, AsA content and CAT activity in control leaves were more than double in the Persian lime compared with the Mexican lime. For Las+S samples, an increase in MDA was observed in Persian limes but did not lead to a change in H_2_O_2_ content compared with Las+AS (Table 5). AsA content decreased in Mexican lime and Persian lime Las+S samples compared with Las+AS samples. On the other hand, for APX, Las+S activity values were higher than for Las+AS for Mexican limes and Persian limes. For CAT, the activity values for Las+S Mexican lime remained stable compared with Las+AS, but the values decreased in Persian limes. Assays for copper (Cu), iron (Fe), and zinc (Zn) ion contents were also performed for the same type of leaf samples (Table 6). An increase in Cu content was observed in Mexican lime and Persian lime Las+S samples compared with controls. The increase is also significant regarding the Fe content in Mexican limes compared with the control and Las+AS and between Las+AS and Las+S. This same tendency was observed in Persian limes but to a lesser extent. Finally, decreases in Zn content were highlighted in Mexican lime and Persian lime Las+S samples compared with the Las+AS and control samples.

**TABLE 5:** Stress biomarkers (MDA, H_2_O_2_), antioxidant molecule (AsA) contents, and detoxification enzyme (APX, CAT) activities in the control (Las−) and infected leaves that were asymptomatic (Las+AS) and symptomatic (Las+S) from Mexican and Persian limes, respectively.

	Las-		Las+AS		Las+S	
			
	Mexican lime	Persian lime	Mexican lime	Persian lime	Mexican lime	Persian lime
**MDA (Arb. unit)**	1 ± 0.15 a	1.15 ± 0.32 a	2.44 ± 0.06 c	1.37 ± 0.50 ac	1.98 ± 0.06 c	3.74 ± 0.36 d
**H_2_O_2_ (Arb. unit)**	1 ± 0.03 a	0.96 ± 0.04 a	2.15 ± 0.08 c	2.04 ± 0.11 c	1.77 ± 0.15 b	2.08 ± 0.04 c
**AsA (Arb. unit)**	1 ± 0.31 a	2.34 ± 0.08 b	3.69 ± 1.59 b	7.85 ± 0.72 c	1.16 ± 0.20 a	2.60 ± 0.38 b
**APX (Arb. unit)**	1 ± 0.69 ab	0.08 ± 0.08 a	1.05 ± 0.38 ab	1.76 ± 0.82 b	4.08 ± 0.10 c	4.74 ± 0.05 c
**CAT (Arb. Unit)**	1 ± 0.6 a	2.9 ± 0.2 b	1.6 ± 0.4 a	15.0 ± 5.7 d	2.4 ± 0.9 b	4.8 ± 0.1 c

*Values of Mexican lime prior infection were used as baseline to adjust the values of Las+AS and Las+S of Mexican lime and Persian lime. Results are expressed as mean + SE (*n* = 4–6). ANOVA tests were performed to determine if HLB led to significant differences. For each given datum, different letters indicate a statistical difference between genotypes (one-way ANOVA followed by Tukey’s post hoc test, *P* ≤ 0.05).*

**TABLE 6:** Cu, Fe, and Zn contents in control (Las−) and infected leaves that were asymptomatic (Las+AS) and symptomatic (Las+S) from Mexican and Persian limes, respectively.

	Las-		Las+AS		Las+S	
			
	Mexican lime	Persian lime	Mexican lime	Persian lime	Mexican lime	Persian lime
**Total Cu (mg/kg)**	3.85 ± 0.96 a	4.92 ± 1.23 ab	2.45 ± 0.61 a	6.82 ± 1.77 bc	5.18 ± 1.30 b	9.05 ± 1.36 c
**Total Fe (mg/kg)**	86.11 ± 4.31 b	71.17 ± 3.56 a	127.74 ± 6.39 d	84.06 ± 4.20 b	253.40 ± 2.53 e	107.47 ± 1.07 c
**Total Zn (mg/kg)**	21.24 ± 1.06 b	27.07 ± 1.35 c	20.30 ± 1.02 b	27.15 ± 1.36 c	10.51 ± 0.53 a	24.18 ± 1.21 c

*Results are expressed as mean + SE (*n* = 4). ANOVA tests were performed to determine if HLB led to significant differences. For each given datum, different letters indicate a statistical difference between genotypes (one-way ANOVA followed by Tukey’s post hoc test, *P* ≤ 0.05).*

## Discussion

### Anatomical and Physiological Differentiations Between Mexican Lime and Persian Lime Cultivars

Phenotypic differentiation associated with polyploidy has been extensively investigated (for review, see Ruiz et al., 2020). Ploidy in citrus leads to greater sizes cell and organ sizes, as observed for the leaf petiole diameters and the leaf surface areas of 4× compared with Mexican limes (Table 4; Mouhaya et al., 2010; Allario et al., 2011). The Persian lime cultivar has higher leaf thickness values and larger stomatal and epidermal cell sizes than Mexican lime leaves do. Similarly, the stomatal density in the Persian lime is significantly lower than in the Mexican lime cultivar (Table 1). These results are in agreement with the results obtained with other triploid and tetraploid plants, as polyploidy induces an increase in leaf thickness, an increase in size, and a decrease in stomatal cell density (Allario et al., 2011; Padoan et al., 2013; Lourkisti et al., 2020). Leaf greenness, intrinsic photosystem II QY, and gas exchange parameters have also shown different photosynthetic activities: Persian limes generally presented higher values of SPAD, QY, *gs*, *A*, and *E* than Mexican limes did. These higher photosynthetic and respiratory functions may explain the greater vigor observed in 3× Persian limes that should be favorable under HLB constraint (Deng et al., 2019). In agreement with these results for Persian limes, Lourkisti et al. (2020) showed a higher photosynthetic efficiency in 3× mandarin hybrids compared with 2× clementine hybrids. Interestingly, the photosynthetic activities of tetraploid seedlings (Allario et al., 2011) and diploid sweet orange grafted on tetraploid rootstocks (Allario et al., 2013) were reduced compared to plants with diploid root systems, in association with higher constitutive ABA biosynthesis in tetraploid roots. It appears that polyploidy in roots and canopies have an antagonist effect on photosynthetic and respiratory function.

### Anatomical and Physiological Characterizations of Mexican and Persian Lime Cultivars Grown Under HLB Stress

Observations in the field showed that the Persian lime trees were much less affected than Mexican lime trees were, which fits with previous observations regarding HLB tolerance (Evans et al., 2014; Deng et al., 2019). Several cultivars, including lemons [(*C. limon (L.) Burm. F*.) and Persian lime] and the “LB8-9” Sugar Belle^®^ mandarin hybrid (“Clementine” mandarin × “Minneola” tangelo; Gmitter et al., 2010) have shown apparent HLB tolerance (Albrecht and Bowman, 2012; Ramadugu et al., 2016; Killiny et al., 2017; Miles et al., 2017; Wang et al., 2017). This tolerance was associated with the maintenance of vigorous growth and fruit yield. In greenhouse conditions, infected Mexican lime leaves were much more yellow than Persian lime leaves. These results are in agreement with the variation of gas exchange between control and infected trees measured in Persian limes and Mexican limes. Triploid Persian limes maintained the highest *g*_*s*_, *A*, and *E* values compared with Mexican limes. For infected trees, the values measured for Mexican limes were low, suggesting that the metabolism of this cultivar was extremely affected. Koh et al. (2012) showed that HLB leads to an increased callose synthesis in the phloem vessels, resulting in clogging of the pores at the sieve plate between cells (Achor et al., 2010). To verify whether the differences between Mexican lime and Persian lime cultivars could be explained by different levels of intercellular sieve pore plugging, we performed SEM on petiole control samples. Phloem cells and sieve plate pores of Persian lime trees were larger than those of Mexican lime trees. This observation can be directly related to the ploidy levels of these two varieties. In HLB-infected petiole of symptomatic leaves, the phloem cell sieve pores were found to be heavily obstructed by callose deposition in the Mexican limes and Persian limes, as previously mentioned (Achor et al., 2010; Koh et al., 2012; Albrigo et al., 2014), indicating that the metabolisms of such leaves were strongly affected for both varieties. However, the phloem cell wall distortion and a sieve element collapse induced by the bacterium (Etxeberria and Narciso, 2015) seemed to be more limited in Persian limes: In that genotype, the petiole diameter and the phloem petiole area were increased, while a strong decrease of these parameters was observed in Mexican lime. Recent work performed by Deng et al. (2019) showed that the HLB-tolerance traits of “Bearss” lemons and “LB8-9” Sugar Belle^®^ mandarins are associated with more effective phloem regeneration, thus limiting the cell wall distortion and sieve element collapse. Their conclusion is in agreement with the increases in petiole diameter, phloem, and petiole cortex area observed in the Persian lime, suggesting that the Persian lime experienced a better phloem regeneration than the Mexican lime, limiting the phloem degradation and collapse. Interestingly, in Las+AS petioles of Persian limes, sieve pores remained open, and limited callose deposits were observed. Contrarily, the sieve pores remained plugged in infected (Las+AS and Las+S) Mexican limes. The higher callose depositions in Las+AS petioles of Mexican limes compared to Persian limes, as observed by SEM, were confirmed by the presence of a strong white ring targeting the callose deposit when using aniline blue. The level of callose deposition is associated with a higher fluorescence labeling of Las in the Mexican lime and correlates with the lower Ct values measured in that genotype compared with the Persian lime, suggesting the faster development of the bacterium and a stronger impact at the physiological level. Starch accumulation is a classical symptom of a HLB-infected tree. A large number of starch grains were visible only in the Mexican lime, emphasizing the stronger impact of HLB in that genotype, as previously observed in different aerial organs (Schneider, 1968; Ed Etxeberria et al., 2009).

**FIGURE 4:**
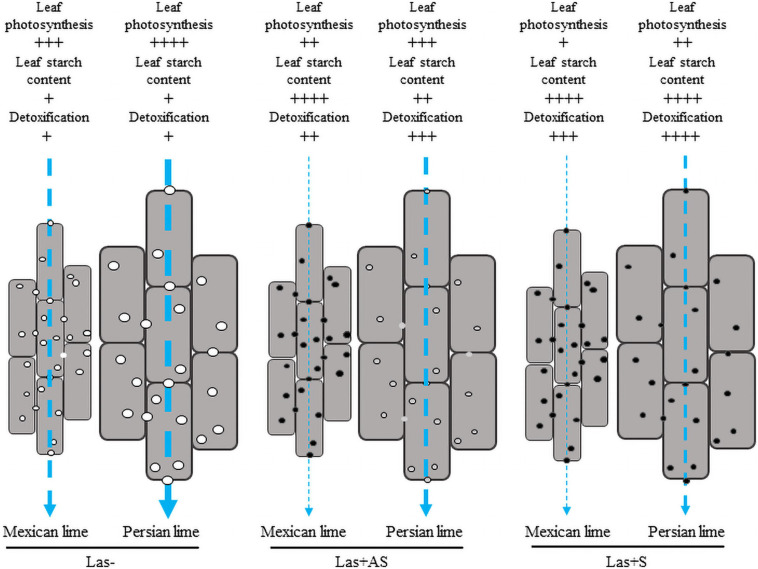
Cellular model reflecting the impact of HLB at the leaf level with regard to phloemic sap flow in the petioles of the control (Las−) as well as infected leaves that were asymptomatic (Las+AS) and symptomatic (Las+S) from Mexican and Persian limes, respectively. Vertical arrows indicate the expected strength of the phloemic sap flow. In the control petiole, the pores and the cells are bigger, and the photosynthetic rate in the Persian lime is higher compared with the Mexican lime. Thus, the phloemic sap flux is expected to be higher in Persian lime than in the Mexican lime, which may favor the growth rate of the Persian lime. In Las+AS leaves, the photosynthetic rate and the detoxification are higher in the Persian lime than in the Mexican lime. The pores in the Mexican lime are clogged, which favors the accumulation of starch in leaf; however, pores are still open in the Persian lime, which helps with maintaining the flow of phloemic sap in that genotype. In Las+S leaves, the photosynthetic rate and the detoxification are still higher in the Persian lime than in the Mexican lime even though all pores are clogged. In both genotypes, a strong increase in the starch content is observed. One may expect, however, that for a given petiole length to cross, the larger size of the cells in the Persian lime corresponds to a more limited number of cell walls of the phloem to cross than in the Mexican lime. Associated with a greater photosynthetic rate, this will help in maintaining a higher phloemic sap flow in Persian lime Las+S than in Mexican lime Las+S.

In Las− leaves of the Mexican lime compared with the Persian lime, a fivefold increase of the starch content (Table 3), a faster plugging of the pores between phloem cells (Figure 3C), and maintaining of lower values of gas exchange parameters (Figure 2) were noted. Taken together, this suggests that the larger cell and pore sizes in the Persian lime leaves and petioles, resulting from polyploidy, favored the longer maintenance period of the intercellular phloem flows in this variety (Figure 4). In turn, this reflects the specific phenology of the Persian lime, with a slower plugging of phloem cell pores and a better phloem regeneration (Deng et al., 2019), in agreement with a longer leaf lifetime of that genotype in comparison with the Mexican lime genotype.

### Differential Response to Oxidative Stress in the Mexican and Persian Limes

Mature fruit drop in HLB-infected citrus trees was shown to be related to oxidative stress genes coding for antioxidant enzymes, which were upregulated in HLB-susceptible citrus compared with tolerant citrus (Tang and Vashisth, 2020). MDA content is considered an indicator of oxidative stress as it corresponds to the final product of lipid peroxidation (Luna et al., 2000; Tabassum et al., 2017). A twofold increase in MDA content was observed in Mexican lime Las+AS leaves compared with the controls, whereas no significant change was observed in the Persian lime leaves at this disease development stage. The measured MDA concentrations suggested that Mexican limes were affected earlier by stress than the Persian limes were. In the Persian lime Las+AS leaves, the unchanged MDA content was consistent with a better HLB tolerance associated with more limited cellular oxidative damage. In triploid citrus subjected to natural chilling (Lourkisti et al., 2020) and to water deficits (Lourkisti et al., 2021), as well as tetraploid citrus seedlings subjected to nutrient deficiency (Oustric et al., 2019) and salinity (Khalid et al., 2020), a lower MDA content was consistent with more limited oxidative damage, suggesting an improved tolerance of polyploid citrus to oxidative stress. In Mexican lime Las+S leaves, a slight decrease in the MDA content was observed, whereas a threefold increase was observed in the Persian lime leaves, suggesting that the bacterium requested a longer amount of time to initiate oxidative stress in polyploidy. If H_2_O_2_ contents were similar in Mexican lime and Persian lime Las−, Las+AS, and Las+S samples, APX and particularly CAT activities were stronger in the Persian lime samples, which, in turn, favored the limitation of oxidative stress in the investigation of triploid citrus subjected to stress (Lourkisti et al., 2020, 2021). CAT, which can convert millions of H_2_O_2_ to water and oxygen per second, is undoubtedly the key marker differentiating the Persian lime from the Mexican lime in terms of ROS balance efficiency at the earlier disease stage. Indeed, the higher AsA content and the higher CAT activity in the Persian lime Las− leaf compared with the Mexican lime leaf, both of which have a similar H_2_O_2_ and MDA content, would be in agreement with the better efficient antioxidant system in polyploid (Figure 4) as previously proposed by Allario et al. (2013).

Ascorbate peroxidases reduces H_2_O_2_ using AsA as the electron donor (Foyer and Noctor, 2005). AsA, with a more than twofold concentration in the Persian lime at the asymptomatic stage, can also act directly with ROS. An increase in Fe content was observed in Las+AS and Las+S leaf samples compared with the control. This increase was even stronger in the Mexican lime compared with the Persian lime (Table 6). HLB reduced foliar concentrations of calcium (Ca), magnesium (Mg), manganese (Mn), zinc (Zn), and iron (Fe) in infected leaves that were highly symptomatic, but HLB did not affect the root concentrations of these same essential nutrients (Hamido et al., 2019; Inoue et al., 2020).

Huanglongbing-polyploidy interactions are contrary to the well-known link between iron content and CAT activity (Lombardi et al., 2003). Indeed, the iron content in the Mexican lime is roughly tripled from Las− to Las+S and is associated with a strong CAT activity increase, but this is not true in the Persian lime. When HLB occurs in the Persian lime, even at the early stage (Las+AS), the CAT increases fivefold, whereas the iron content is close to that of the control.

The Fe and Cu increase that we observed in Las+AS and Las+S was probably transient and would cause the plant to limit HLB-induced stress through the translocation of these important micronutrients from the root to the leaf. Indeed, a foliar application of Fe^2+^ was shown to relieve the symptom of HLB in citrus trees (Inoue et al., 2020). Also, the decrease observed in Zn contents from Las+ to Las+AS and Las+S may suggest that the Fe, Cu, and Zn decreases induced in strongly infected leaves do not happen at the same time. In association with iron (Fe^2+^), H_2_O_2_ can give rise to high deleterious radical species: hydroxyl radicals (Migdal and Serres, 2011; Inagaki et al., 2016). Indeed, the Fenton reaction induced by using Fe^2+^ + H_2_O_2_ would favor the production of OH. Maintaining a relatively low level of H_2_O_2_ relates to the reduction of H_2_O_2_ through APX and its use in the Fenton reaction.

## Conclusion

In the field, the triploid Persian lime is one of the most HLB-tolerant cultivars. We have shown that this variety behaves better at a physiological and a biochemical level than its diploid Mexican lime parent does. Anatomical, physiological, and biochemical differentiations due to ploidy variation explain a large part of the behavior of the Persian lime under an HLB constraint. Using SEM analysis, we observed that the Persian lime’s better tolerance to HLB was associated with the larger pore size in the sieve plate of the phloem cells of the leaf petiole compared with the Mexican lime. In addition, the investigation of infected petiole samples of asymptomatic leaves showed much larger callose depositions onto the Mexican lime versus the Persian lime, whereas symptomatic leaves showed important depositions onto Mexican lime and Persian lime pores. Our results provide insights into specific traits associated with polyploidy, such as the size of the pores of the phloem, as well as the Persian lime’s detoxification processes, which can help maintain the phloemic flow in the plant and, thus, result in a better HLB-tolerance (Figure 4). Thus, investigations are required to decipher the molecular determinants of Persian lime cultivars’ better HLB tolerance.

## Data Availability Statement

The original contributions presented in the study are included in the article/Supplementary Material, further inquiries can be directed to the corresponding author.

## Author Contributions

GS, LC, SB, and RB-N performed the experiments and collected the physiological data. GS, PB, PO, and RM performed the statistical analyses, interpreted the results, and drafted the manuscript. GS and OG performed the analysis by SEM. LC and OG helped to draft the manuscript. All authors contributed to the article and approved the submitted version.

## Conflict of Interest

The authors declare that the research was conducted in the absence of any commercial or financial relationships that could be construed as a potential conflict of interest.

## Publisher’s Note

All claims expressed in this article are solely those of the authors and do not necessarily represent those of their affiliated organizations, or those of the publisher, the editors and the reviewers. Any product that may be evaluated in this article, or claim that may be made by its manufacturer, is not guaranteed or endorsed by the publisher.

## Footnotes

1
http://rsbweb.nih.gov/ij/

2
www.systatsoftware.com

## Supplementary Material

Supplementary Figure 1 Fish analysis using a probe targeted against Las performed by using the same amount of grounded petiole material of the control (Las−; **A,B**) as well as infected leaves that were asymptomatic (Las + AS; **C,D**) and symptomatic (Las + S; **E,F**) from the Mexican and Persian limes, respectively. White arrows indicate the florescence resulting from the presence of Las. Bars = 12 μm.
