# Supplementary figures and images for: Specific Physiological and Anatomical Traits Associated With Polyploidy and Better Detoxification Processes Contribute to Improved Huanglongbing Tolerance of the Persian Lime Compared With the Mexican Lime
Source: Front Plant Sci. 2021 Aug 26;12:685679. doi: 10.3389/fpls.2021.685679 (PMC8427660; doi:10.3389/fpls.2021.685679)

## Slide 1
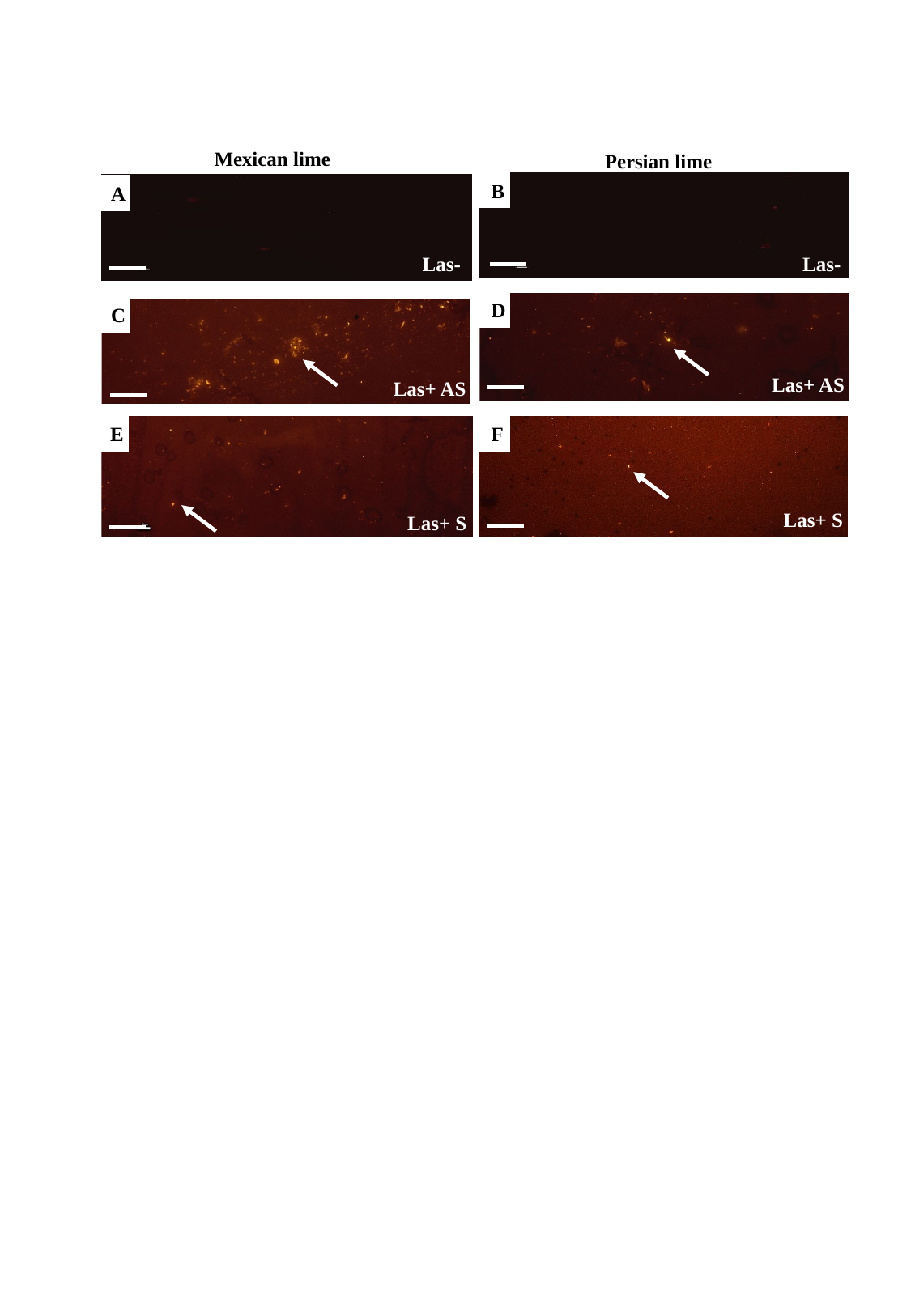

Mexican lime
Persian lime
B
A
D
C
F
E
Las-
Las-
Las+ AS
Las+ AS
Las+ S
Las+ S

Supplement: Supplementary Figure 1 — Fish analysis using a probe targeted against Las performed by using the same amount of grounded petiole material of the control (Las−; A,B) as well as infected leaves that were asymptomatic (Las + AS; C,D) and symptomatic (Las + S; E,F) from the Mexican and Persian limes, respectively. White arrows indicate the florescence resulting from the presence of Las. Bars = 12 μm. [file Presentation_1.PPTX]
